# Leptin and Associated Mediators of Immunometabolic Signaling: Novel Molecular Outcome Measures for Neurostimulation to Treat Chronic Pain

**DOI:** 10.3390/ijms20194737

**Published:** 2019-09-24

**Authors:** Thomas M. Kinfe, Michael Buchfelder, Shafqat R. Chaudhry, Krishnan V. Chakravarthy, Timothy R. Deer, Marc Russo, Peter Georgius, Rene Hurlemann, Muhammad Rasheed, Sajjad Muhammad, Thomas L. Yearwood

**Affiliations:** 1 Department of Neurosurgery, Division of Functional Neurosurgery and Stereotaxy, University of Erlangen-Nueremberg, 91054 Erlangen, Germany; Michael.Buchfelder@uk-erlangen.de; 2 Departmen of Basic Medical Sciences Shifa College of Pharmaceutical Sciences, Shifa Tameer-e-Millat University, Islamabad 44000, Pakistan; shafqatrasul@yahoo.com; 3 Department of Anesthesiology and Pain Medicine, University of California, San Diego, CA 92161, USA; kvchakravarthy@UCSD.EDU; 4 VA San Diego Healthcare System, California, San Diego, CA 92161, USA; 5 The Spine and Nerve Center of the Virginias, Charleston, WV 25301, USA; doctdeer@aol.com; 6 Hunter Pain Clinic, 2300 Newcastle, Australia; algoguy@gmail.com; 7 Nambour Selangor Private Hospital, Nambour, 4560 Queensland, Australia; painrehab@netspace.net.au; 8 Department of Psychiatry, Division of Medical Psychology, University Hospital Bonn, 53127 Bonn, Germany; rene.hurlemann@ukb.uni-bonn.de; 9 Department of Neuro-Urology Marien Hospital Herne, University Clinic of the Ruhr University Bochum, 44625 Herne, Germany; muhammad.rasheed@ruhr-uni-bochum.de; 10 Department of Neurosurgery, University Hospital Duesseldorf, 40225 Düsseldorf, Germany; msajjad51@yahoo.com; 11 Neuromodulation Specialists LLC, Fairhope, AL 36532, USA; nopaindr@mac.com

**Keywords:** chronic pain, immunometabolism, systemic inflammation, neuromodulation, biomarkers development, quantitative outcome measures

## Abstract

Chronic pain is a devastating condition affecting the physical, psychological, and socioeconomic status of the patient. Inflammation and immunometabolism play roles in the pathophysiology of chronic pain disorders. Electrical neuromodulation approaches have shown a meaningful success in otherwise drug-resistant chronic pain conditions, including failed back surgery, neuropathic pain, and migraine. A literature review (PubMed, MEDLINE/OVID, SCOPUS, and manual searches of the bibliographies of known primary and review articles) was performed using the following search terms: chronic pain disorders, systemic inflammation, immunometabolism, prediction, biomarkers, metabolic disorders, and neuromodulation for chronic pain. Experimental studies indicate a relationship between the development and maintenance of chronic pain conditions and a deteriorated immunometabolic state mediated by circulating cytokines, chemokines, and cellular components. A few uncontrolled in-human studies found increased levels of pro-inflammatory cytokines known to drive metabolic disorders in chronic pain patients undergoing neurostimulation therapies. In this narrative review, we summarize the current knowledge and possible relationships of available neurostimulation therapies for chronic pain with mediators of central and peripheral neuroinflammation and immunometabolism on a molecular level. However, to address the needs for predictive factors and biomarkers, large-scale databank driven clinical trials are needed to determine the clinical value of molecular profiling.

## 1. Introduction

The estimated prevalence of chronic pain varies between 20–40% and is characterized by an increased socioeconomic burden negatively affecting both the physical condition and quality of life of patients [1]. Chronic pain conditions pose very high costs of treatment worldwide. The estimated costs (National academic press, USA) of pain treatments ranged between US$ 560–635 billion in 2010. Clearly, the recent opioid crisis demonstrated the need to replace former treatment strategies for chronic pain disorders with non-opioid pharmacological paradigms and neurostimulation therapy.

Chronic pain, as a consequence of tissue injury, is characterized by activation and sensitization of central and peripheral pain pathways mediated through multiple inflammatory molecules including leptin, its relatives adiponectin and ghrelin, interleukin (IL) IL1-β, tumor necrosis factor TNF-α, IL-6, high-mobility group box-1 HMGB-1, damage-associated molecular pattern DAMP´s, oxytocin, and multiple chemokines, reactive oxygen species, ATP, lipids, and vasoactive amines. Neuropathic pain is initiated by a lesion in the somatosensory nervous system, is strongly associated with neuroinflammation, and is considered a more severe form of pain that also influences sleep quality and psychological functioning [2]. Experimental studies suggest that these inflammatory peptides including adipocytokines interact with neural transmission by promoting hyperexcitability in neural circuits relevant for the development of chronic pain disorders (e.g., spinal cord dorsal column, dorsal root ganglia, and trigeminal ganglion). For instance, Il-1 β and leptin (pro-inflammatory) and oxytocin (anti-inflammatory) exert their effects through specific receptors located either at the terminal C-fibers (neuropathic pain) or the trigeminal ganglion (migraine). Pharmacological treatment with a variety of classes of drugs and behavioral therapy are first-line treatments for neuropathic pain [3,4,5,6,7,8], but many patients fail to experience clinically meaningful pain relief from these treatments alone. Drug resistance and loss of efficacy in chronic pain treatment are perhaps due to the involvement of multiple disturbed physiological and psychological interrelated pathways that contribute to the severity of chronic pain. Hence, understanding mechanisms of relevant comorbidities underlying chronic pain (e.g., metabolism, depression, and anxiety) may help to develop novel diagnostic and treatment options. In addition to their role in the transition and maintenance of chronic pain, most of the described mediators have been associated with a broad range of chronic disorders (neurological, psychiatric, cardiovascular, and metabolic) due to their multifunctional characteristics, which in turn may explain the clinically observed co-occurrence of the so-called comorbidities [9,10,11,12]. Spinal and peripheral neurostimulation therapies have repeatedly shown meaningful pain relief in recent studies to treat various chronic pain disorders (failed back surgery, complex regional pain syndrome (CRPS), and migraine) targeting the spinal cord, the dorsal root ganglia (DRG), and peripheral nerves (vagus nerve stimulation; VNS) [3,4,5,6,7,8]. The strongest rationale for considering neurostimulation approaches to treat chronic pain is the growing understanding that all forms of pain are ultimately the result of an interplay of central and peripheral properties of the nervous system, which in turn allows neurostimulation therapies to interact with inflammatory pain signaling [13,14,15,16,17,18]. Although psychometric alterations (depression, cognitive decline, and anxiety) and functional parameters such as sleep or metabolic state have been reported to interact with neurostimulation responsiveness, objective and quantitative outcome measures on a molecular level remain largely under-investigated. The majority of published studies assess neurostimulation outcomes using subjective score-based tools [18]. In this narrative review, we provide experimental evidence and describe the current state of in-human studies assessing possible relationships between the responsiveness of neurostimulation therapies for chronic pain and molecular profiling of circulating mediators relevant for functional metabolic homeostasis including leptin and its relatives adiponectin and ghrelin, and other associated mediators of metabolic-related inflammation.

## 2. Methods

Search strategies encompassed published literature identified through searches of PubMed, MEDLINE/OVID, and SCOPUS and manual searches of the bibliographies of original and review articles from 2000–2019 with the following terms: chronic pain disorders, systemic inflammation, immunometabolism, adipokines, cytokines, chemokines, biomarkers, metabolic disorders, neuromodulation for chronic pain, and in-human studies to identify primary outcome measures using molecular profiling of inflammation. The primary criteria for inclusion in our review were the following terms: chronic pain disorders—neurostimulation therapies—human studies—inflammatory phenotyping.

## 3. The Role of Circulating Inflammatory Mediators in Chronic Pain Development

**Scheme 1:**
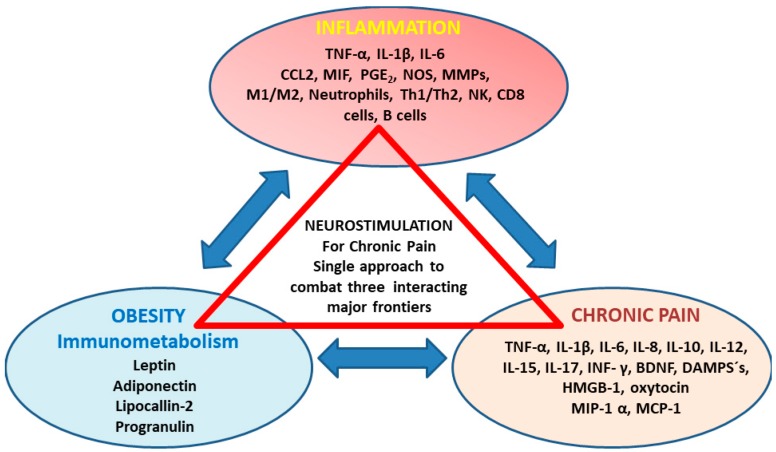
Mediators of inflammation and immunometabolism determined in human neurostimulation studies for chronic pain [8,9,10,27,28,29,30,31,32,33].

Cytokine and chemokine mediated inflammation is an essential aspect of the pathophysiological processes of pain. Evidence from clinical and pre-clinical data support the notion that pro-inflammatory cytokines including IL-1β, TNF-α, and chemokines such as monocyte chemotactic protein-1 (MCP-1) directly modulate cell activity in different types of neural cells both in the peripheral and central nervous systems (PNS and CNS respectively) [19,20,21,22,23]. In the PNS, topical application of TNF-α to the peripheral axons evoked abnormal spontaneous activity from nociceptive neurons [20]. In a similar manner, TNF-α application to the somata of the DRG neurons *in vitro* provoked abnormal spontaneous activity. Moreover, large myelinated fast-conducting Aβ neurons specifically are excited by topical application to the DRG of TNF-α or by an autologous herniated nucleus pulposus extract [21]. Interestingly, TNF-α can enhance the sensitivity of sensory neurons to the excitation produced by capsaicin, and this enhancement likely is mediated by the neuronal production of prostaglandins [20]. Mechanistically at the level of cellular signals, TNF-α-induced neuronal excitation is mediated by the cAMP-dependent protein kinase (PKA) pathway [20]. The p38 mitogen-activated protein kinase (MAPK) is also involved in TNF-α-induced cutaneous hypersensitivity to mechanical or thermal stimulation [24]. Interestingly, the data from IL-6 knockout mice indicate that IL-6 plays a facilitating role in sympathetic sprouting induced by nerve injury, and its effect on pain behavior is promoted through sympathetic sprouting in the dorsal root ganglia [25]. Consistent with the above data, localized inflammation of the DRG up-regulates a number of pro-inflammatory cytokines including IL1-β, IL-6, TNF-α, HMGB-1, DAMP´s, oxytocin, multiple chemokines, reactive oxygen species, ATP, lipids, and vasoactive amines induces abnormal sympathetic sprouting in the absence of peripheral nerve injury [26]. It suggests a possible correlation between inflammatory responses and sympathetic sprouting, which are two well-known mechanisms implicated in various chronic pain states. This data taken together shows, pro-inflammatory cytokines to be involved in the pathophysiology of the development of inflammatory and neuropathic pain with intense sympathetic nervous system involvement. Hence, specific cytokines and their neutralizing antibodies could be utilized locally or systemically to block pro-inflammatory cytokines for the treatment of chronic pain. These specific antibodies against pro-inflammatory cytokines would act to disrupt the hyperexcitability cycle taking place in the sensory neurons and hence may provide a new, non-NSAR, non-opioid therapeutic approach for the treatment of pathological pain due to inflammation or peripheral nerve injury. Neurostimulation therapies (non-invasive–invasive) targeting different neural targets are successfully being used in clinical pain medicine, but their mode of action and their interaction with cellular and molecular properties of the neuro-immune axis in humans has been poorly investigated, but would probably have a considerable diagnostic and therapeutic yield for pharmacological and non-pharmacological (neurostimulation) approaches (Scheme 1).

### 3.1. Possible Associations between Back Pain (Spino-Nociceptive Traffic) with Immunometabolism

Experimental and in-human studies indicate that obesity and associated diseases such as diabetes or cardiovascular disorders (heart failure, stroke, and cerebral ischemia) can be regarded as a consequence of ongoing chronic inflammation. However, both preclinical and human trials have often been contradictory in assessing the relationships between low back pain and chronic inflammation [27,28,29,30,31,32]. In part, metabolic disorders and related comorbidities have gaped to the development of low back pain indicative of an imbalanced brain–immune communication, which could promote central and peripheral inflammation [32,33,34]. The complex interplay of pro- and anti-inflammatory cytokines (e.g., IL-6, IL-10, IL-13, tumor-necrosis factor (TNF-α), and oxytocin) and other pro- and anti-obesity-relevant peptides, especially leptin (LP), adiponectin (AN), and ghrelin (GH), are thought to evoke the genesis and maintenance of both chronic conditions (pain–metabolic disorder) [28,32,33,34,35,36,37,38,39]. Notably, immunoregulatory IL-10, TNF-α, and IL-1 have been linked to the pathophysiology of multiple chronic disorders including pain, obesity, diabetes, metabolic syndrome, and depression/anxiety, most of which are frequently observed clinically [6,7,34,40].

In this context, a principal member of the adipokine superfamily, leptin (LP), derived from adipocytes, vascular smooth muscle cells, and cardiomyocytes, represents one of the key players in favor of an obese state with studies supporting the relationship between LP expression and body fat mass [34,41,42]. Contrary to IL-10, elevated LP concentration may predict the development of obesity, myocardial ischemia, heart failure, and insulin resistance on the one hand. Clearly, chronic pain is fraught frequently with obesity, but not in general; hence leptin and relatives should be integrated into chronic pain assessment on the other hand as LP drives IL-1 synthesis, which in turn promotes a chronic pain state [34]. Contrary, decreased concentrations of adiponectin (AN), another member of the adipokine family and a neuropeptide solely synthetized by adipocytes, has been suspected of inducing metabolic disorders (e.g., type 2 diabetes, arterial hypertension, and coronary artery disease). Whether concentration changes in circulating AN relate to changes in body weight (body mass index; BMI) or only represent an epiphenomenon is still largely unknown [4,5,6,41,42,43]. AN affects the resistance of insulin receptors (anti-diabetic effect), lipid oxidation, vasodilatation, and anti-arteriosclerosis effects [34,41,42]. To generate an estimated risk profile for metabolic disorders, some studies used the LP–AN ratio (LAR) rather than LP or AN quantification alone. The neuropeptide ghrelin (GH) promotes lipolysis and protects the vascular endothelial architecture (vasoconstriction-suppression) effects. GH is synthesized in the stomach and binds to receptors in the central hypothalamus to stimulate the appetite. Clinical studies have indicated that GH levels are positively correlated with body fat mass. Decreased GH concentrations were detected in obese individuals compared to non-obese controls. Thus, the relationships between chronic low back pain, a very common spinal-nociceptive issue, and immune-driven metabolic disorders appear to be intricately intertwined [44,45,46,47,48,49].

### 3.2. Possible Associations between Migraine (Trigemino-Nociceptive Signaling) with Immunometabolism

There is increasing evidence that migraine, a neurological disorder of the trigeminal-nociceptive system, is associated with the contribution of adipose tissue cellular components of systemic inflammation. Circulating mediators of inflammation involved in the mechanisms of migraine pathology are secreted from adipocytes and adipose tissue-derived immune cells [4]. It is widely recognized, white adipose tissue (WAT), an endocrine-active organ, not only functions as a site of energy storage but also has the capability to exacerbate or reduce systemic inflammation. Within WAT there is a complex interplay between adipocytes involving the balance of synthesis of LP and AN, and the neuro-immune axis to essentially establish physiological homeostasis which, if dysfunctional, leads towards obesity and drives cascades of neuroinflammatory mediators that impact many of the neural circuits relevant to migraine [50,51]. LP (pro-inflammatory), a metabolic marker produced by WAT cells drives COX-2 related pathways of IL-1β in glial cells and neurons of the hypothalamic–pituitary axis. Additional experimental work demonstrated that ghrelin improved light sensitivity, an indicator for migraine-related autonomic features, and evoked behavioral changes in an experimental animal model of head pain [51].

### 3.3. Oxytocin at the Crossroad of Trigeminal-Spinal Pain Transmission and Immunometabolism

Preclinical as well as human studies indicate that the neuropeptide oxytocin impacts pain perception and processing of both the trigemino-nociceptive (e.g., migraine) and the spino-nociceptive (neuropathic pain) systems [52,53,54,55,56,57,58,59,60,61,62,63,64,65,66,67,68]. In addition, oxytocin interacts with metabolic neural circuits and metabolic-related organ systems (liver, muscle, adipose tissue, and pancreas) [53]. The magnocellular neurons located in the hypothalamic nuclei PVN (nucleus paraventricularis) and the SOP (supraoptic nucleus) synthesize oxytocin, which is transmitted in vesicles via anatomical projections to the posterior pituitary lobe, the amygdala, the hippocampus, and the cerebral cortex, ultimately promoting oxytocin release into the systemic blood circulation. A second population of parvocellular oxytocinergic neurons in the PVN projects directly to the brainstem and the spinal cord (dorsal column layers–dorsal root ganglion) without the involvement of the systemic blood circulation [54,55,56]. Based upon available evidence, oxytocin appears to negatively impact nociceptive transmission and signaling in all these areas directly, via on-site neuronal delivery, or indirectly, via the blood circulation, thereby regulating both central and peripheral inflammatory processes.

**Scheme 2:**
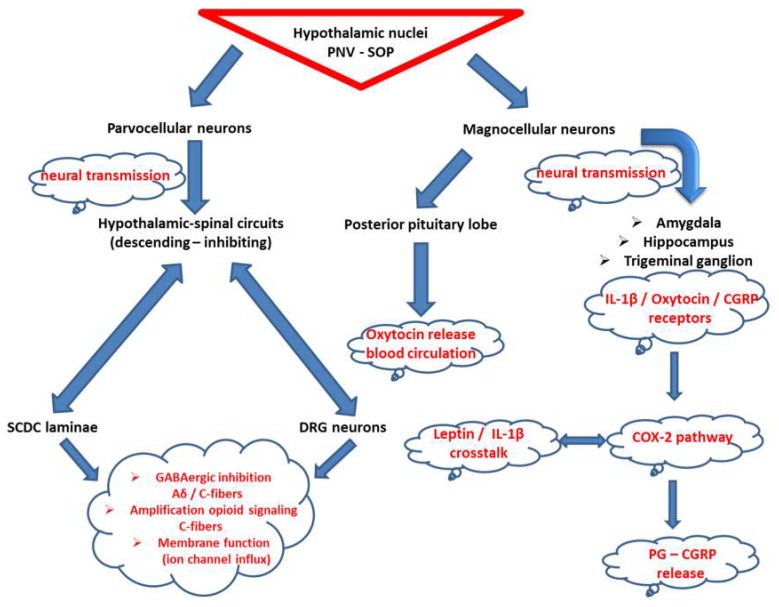
Oxytocinergic descending hypothalamic-spinal circuit promotes anti-nociception and analgesia. Oxytocin is synthesized in neurons located within the hypothalamic nuclei (nucleus paraventricularis of the hypothalamus; PVN) and the supraoptic nucleus (SOP). Magnocellular neurons are distributed in the PVN and in the SOP and project to the posterior pituitary lobe, where oxytocin is released into the systemic blood circulation and impact neural transmission of the amygdala and hippocampus. Parvocellular oxytocinergic neurons (PVN) release oxytocin in the brainstem, spinal cord, and trigeminal ganglion [5,52,54,55,56,57,58]. Abbreviations: PNV—nucleus paraventricularis; SOP—supraoptic nucleus; SCDC—spinal cord dorsal column; DRG—dorsal root ganglion; GABA—gamma aminobutyric acid; CGRP—calcitonin-gene related peptide; COX—cyclooxygenase; PG—prostaglandine.

Experimental animal models and in-human studies have shown positive analgetic effects of oxytocin on cephalgia. After administration of radiographic labeled oxytocin, high concentrations were recorded in the trigeminal nucleus caudalis, the trigeminal ganglia, and the trigeminal branches [57,58]. In addition, several uncontrolled cohort trials observed a meaningful head pain reduction in migraineurs after oxytocin administration. So far, evidence suggests oxytocin may be involved in central sensitization and autonomic dysregulation, conditions frequently observed in migraine phenotypes [59,60,61]. As mentioned, oxytocin projects directly via descending neural routes to specific neuronal cell layers of the spinal cord (dorsal horn laminae) and in addition, is able to bind to receptors of the dorsal root ganglia neurons (DRG) (Scheme 2). This may explain the anti-nociceptive effects of oxytocin in spino-nociceptive circuits elated to pain processing [5,6,7,64,65,66,67,68].

Metabolic disorders frequently co-occur in chronic pain, independently of the pain origin, leading to the question of what role oxytocin may play in metabolic hemostasis under chronic pain conditions. Currently, the hypothalamic–pituitary axis has been recognized as a potential target to restore metabolic function and to impact chronic pain-associated neural transmission, either directly (neuronal) or indirectly (systemic blood circulation). In particular, this becomes an important issue, if clinical phenotypes (pain-metabolism) become difficult to distinguish on a molecular level. It appears that both common and different pathways exist, but this notion is still to be elucidated. Consequently, clinical neurostimulation studies should seek to incorporate metabolic-related markers with the potential to predict chronic pain by diagnostic means. For instance, intranasal administered oxytocin reduced weight, improved insulin sensitivity, and increased pancreatic responsivity in small-scale studies [53]. However, in opposition to what is seen in human trials with apparent conflicting findings, experimental animal studies suggest that oxytocin impacts glucose uptake and lipid utilization in muscle and adipose tissue. This suggests a deterioration in oxytocin signaling may exacerbate obesity and related vascular disorders. 

## 4. Clinical Implications for Neurostimulation Therapies Targeting Chronic Pain Disorders

Possible pathways through which central and peripheral neuromodulation may impact circulating markers of immunometabolism are largely under-investigated in clinical neuromodulation trials. These mechanisms are not likely to be specific for single cytokines, but more likely help control inflammation in quite diverse inflammatory disorders, by activating multiple neuronal networks to control immune cells and thereby minimize the detrimental effects of excessive inflammation seen in chronic pain, dysfunctional metabolism, stroke, epilepsy, and psychiatric disorders. However, the mechanisms of inflammatory action of the applied central and peripheral electrical stimulation modalities (e.g., SCS, DRG, VNS, non-invasive, and invasive brain stimulation) have yet not been fully clarified. A large number of experimental VNS studies have been published which indicate VNS controls the release of multiple cytokines from peripheral macrophages through the neuro-inflammatory reflex involving the nicotinic acetylcholine receptor (nACh) α7. The neuro-inflammatory reflex is in part orchestrated by the dynamic and complex interaction of pro- and anti-inflammatory mediators (e.g., IL-1β, IL-6, IL-8, IL-10, IL-13, interferon-g (IFN-g), TNF-α, and HMGB-1 protein) [69,70,71,72,73]. Combined electrical stimulation of the sciatic nerve and the vagal nerve was found to increase plasma levels of circulating oxytocin as well as touch and pinch indicating the potential of targeted neurostimulation to evoke changes in molecular circuits relevant for pain and metabolism [71]. Given these facts, VNS has been applied for a variety of inflammatory pain disorders (primary headache disorders, rheumatoid arthritis, chronic back pain, neuropathic leg pain, complex regional pain syndrome, chronic pelvic pain, and fibromyalgia) [74].

## 5. In-Human Chronic Pain-Neurostimulation Studies Addressed to Metabolic Molecular Inflammatory Phenotyping (Cervical Non-Invasive VNS, Tonic and BurstDR SCS, DRG-SCS)

To date, there exist a few randomized-controlled and uncontrolled observational neurostimulation human studies, which have quantified peripheral circulating markers of inflammation related to metabolic homeostasis in chronic pain patients [4,5,6,7,75,76,77,78,79] (Table 1).

Most recently, a randomized sham-controlled VNS migraine study [4] found most of the inter-ictal measured cytokines including IL-6, TNF-α, and HMGB1 unchanged before and after VNS as were the metabolic markers leptin, ghrelin, and adiponectin, although serum concentrations were higher in migraine subjects compared to healthy controls. Pro-inflammatory IL-1β plasma concentrations were higher in the sham-VNS group versus the verum nVNS, whereas anti-inflammatory cytokine IL-10 was elevated in migraine patients as compared to healthy subjects but was suppressed three months post-stimulation in both sham and VNS group. These effects were accompanied by a significantly reduced number of severe attacks per month in the nVNS group. Notably, the average BMI was below 25 m^2^/kg, yet nearly 45% of the study cohort suffered from a metabolic-associated disorder (hypertension, cardiac disease) [4]. In a different recent study, significantly increased saliva levels of anti-inflammatory oxytocin and pro-inflammatory IL-1β were detected in the saliva of migraine patients treated before and after VNS treatment, while obesity was present in only 30% of the cohort [52]. In another study including solely healthy subjects, VNS significantly reduced serum IL-1β, TNF- α, IL-6 IL-8, macrophage inflammatory protein [MIP]-1α, and monocyte chemoattractant protein [MCP]-1 demonstrating the anti-inflammatory impact of VNS [80].

Other observational studies and randomized, double-blinded sham-controlled trials have assessed the influence of SCS using tonic waveforms on inflammatory molecular profiles targeting cerebrospinal fluid or skin blister fluid in patients with chronic nociceptive low back and neuropathic leg pain. Inflammatory measures included vascular endothelial growth factor (VEGF), brain-derived neurotrophic factor (BDNF), monocyte chemotactic protein-1 (MCP-1), IL-2, IL-6, IL-12, IL-15, IL-17, TNF-α, interferon-γ (INF- γ), and cellular ratio (Th1-Th2), of which some were changed after conventional, low-frequency SCS (tonic waveform). Although leptin and related metabolic markers were not included, nor were a systematic assessment of weight and/or the existence of metabolic disease performed, some of the measured cytokines and chemokines have been noted in the context of metabolic diseases and depression [75,76,77,78].

In non-obese, failed-back-surgery syndrome patients (FBSS) treated with BurstDR, circulating anti-inflammatory IL-10 was significantly increased after stimulation, while leptin was significantly higher at the baseline with a trend towards lower levels after stimulation. Adiponectin and ghrelin remained unchanged before and after stimulation compared to the healthy control group. Although the study subjects were classified as non-obese according to the BMI, diabetes, hypertension, and cardiovascular disorders were highly prevalent among those subjects with depressive symptoms and disturbed sleep [6,7].

In complex regional pain syndrome (CRPS) and associated comorbidities, a pro-inflammatory state driven by different mediators which results in the complex phenotypes of CRPS may present. In a study of unilateral, targeted dorsal root ganglion stimulation (spine level L-4), improvements were noted in neuropathic pain intensity, sleep quality, and mood in pre-obese CRPS subjects (BMI 29 ± 5.6 kg/m^2^). The choice to study unilateral selective L4 DRG was based on the fact that the knee sensorial innervation is made mainly by this spinal nerve. We believed that if L3 or L5 DRGs were also added, it would have been more difficult to achieve a more precise change, but could not exclude the fact that multisegmental DRG-stimulation would have promoted different changes. Each study subject in this study was noted to have one or more of the following immuno-metabolic clinical disorders: hypertension, diabetes, or cardiac ischemia. On a molecular level, significantly increased serum concentrations of leptin, high-mobility group box 1 (HMGB-1), TNF- α, and IL-6 were quantified before and after L4-DRG stimulation, while IL-1β was significantly increased pre-stimulation, but not after L4-DRG stimulation, and pre-stimulation levels of increased IL-10 were noted to significantly decrease after DRG stimulation. Adiponectin and ghrelin did not differ between CRPS patients and healthy controls at baseline and follow-up [5]. In an extended subgroup assay, the gene expression profile revealed several significantly up- and down-regulated genes. Despite their function for inflammatory host response and neural nociceptive excitability and traffic, some of the genes assessed from the CRPS patients like free fatty acid receptor 2 (FFAR2); interleukin receptor 1 antagonist (ILRN), interleukin 17-F (IL-17F); phospholipase A2, group IIA (PLA2G2); and NADPH oxidase (NOX1) have been associated with metabolic function such as glucose hemostasis, response to starvation, insulin resistance, lipid catabolic function, angiogenesis, blood pressure, and metabolic functions [79]. With regards to CRPS, caution is needed due to the protean nature of CRPS over time and varying phenotypes. CRPS patients remain unique in their presentation and their clinical features. Depending on the time relationship within the inflammatory process, a specific immunological target can change its role by transitioning from the innate to the adaptive immune response. According to the phenotype, the immunological target can vary in role, being either a critical key link in the initiation of the disease or be an inducible marker as the disease progresses associated with a broad variety of physical features such as trophic skin, vascular changes to metabolic factors, bone turn over, trophic skin changes to dystonia [81]. 

This leads to the conclusion that neuromodulation approaches (waveforms) may need to be different (tonic, burst, frequency-regulated) at different times depending upon the effects it can have on chronic pain and relevant comorbidities in balancing the neuro-immune axis (Scheme 1).

## 6. Conclusions and Future Targeted Research

Beyond doubt, this narrative review did not cover the entire spectrum of molecular mediators involved in immunometabolism and inflammation but rather attempted to provide a comprehensive review of inflammatory markers assessed in clinical human neurostimulation trials for chronic pain. Obviously, the pathophysiology of the chronic pain disorders (migraine, CRPS, CLBP, and FBSS) and the approached neural target reported in our review differ fundamentally but may have some overlap in relevant comorbidities such as metabolic disease. In view of the provided data, it appears that molecular profiling of circulating markers of immunometabolism has been under-appreciated and deserves enhanced clinical research attention in the study of patients with chronic pain being treated with neurostimulation therapy. The broad variety of clinical phenotypes observed in chronic pain syndromes and associated comorbidities, of which metabolic-associated disorders are one major representative, points to the urgent need of translational neurostimulation research considering leptin and its related biomarkers of immunometabolism, adiponectin, and ghrelin along with molecular and cellular promoters of neuroinflammation. Hence, a multiplex analysis should be considered via both flow cytometry and mass cytometry techniques to properly determine both extracellular and intracellular changes. These approaches may be complemented by computational tools (computational neurosciences) [82].

To address these translational issues, randomized-controlled neurostimulation trials for chronic pain using a standardized pre-analytic protocol (sample storage, assay, and processing techniques), considering potential bias (age, gender, epigenetic/genetic, and environmental factors) and accompanied by experimental studies are highly warranted.

## Figures and Tables

**Table 1:** In-human neurostimulation trials for chronic pain disorders addressed to molecular phenotyping, including markers relevant for immunometabolism.

Year/Study Design	Pain Disorder	Stimulation Target	Patients	Score-Based Parameter	Stimulation Paradigm	Immunometabolic Parameter	Treatment Duration
**2019/RCT [4]**	Migraine	Cervical branch vagus nerve (nVNS)	[48]	Head pain Severity-Frequency, BMI, PSQI, MIDAS, BDI	120 sec dose bilateral vagus nerve applied 2 times/day (1 ms bursts of 5 kHz sine waves every 40 ms (25 Hz))	ELISA serum (leptin, ghrelin, adiponectin, IL-1β, IL-6, IL-10, TNF-α, HMGB-1)	2 months
**2019/pPS [52]**	Migraine	Cervical branch vagus nerve (nVNS)	[24]	Head pain Severity -Frequency, BMI, PSQI, MIDAS, BDI, EQ-5D-5L	120 sec dose bilateral vagus nerve applied 2 times/day (1 ms bursts of 5 kHz sine waves every 40 ms (25 Hz))	ELISA saliva (IL-1β – oxytocin)	10 weeks
**2019/pPS [5]**	CRPS	DRG (L4-DRG_STIM_)	[24]	Neuropathic pain Severity, BMI, PSQI, BDI	Chronic stimulation, bipolar, 20 Hz, 200-300 µsec, 300–1600 µA	ELISA serum-saliva (leptin, ghrelin, adiponectin, IL-1β, IL-6, IL-10, TNF-α, HMGB-1, BDNF, oxytocin)	3 months
**2019/pPS [79]**	CRPS	DRG (L4-DRG_STIM_)	[12]	Neuropathic pain Severity, BMI, PSQI, BDI	Chronic stimulation, bipolar, 20 Hz, 200–300 µsec, 300–1600 µA	Gene expression blood cells (FFAR2, ILRN, IL-17F, PLA2G2, NOX1 (metabolic function))	3 months
**2018/pPS [6]**	FBSS CLBP	Spinal Cord dorsal column level Th10-11	[24]	FBSS-CLBP Severity, BMI, PSQI, BDI	BurstDR chronic stimulation, bipolar, 40 Hz burst rate, 500 Hz intraburst rate, 1 msec, 2.05–2.45 mA	ELISA-serum (leptin, adiponectin, ghrelin)	3 months
**2017/pPS [7]**	FBSS CLBP	Spinal Cord dorsal column level Th10-11	[24]	FBSS-CLBP Severity, BMI, PSQI, BDI	BurstDR SCS chronic stimulation, bipolar, 40 Hz burst rate, 500 Hz intraburst rate, 1 msec, 2.05–2.45 mA	ELISA-serum (IL-1β, IL-10, TNF-α, HMGB-1)	3 months
**2017/RCT [78]**	CRPS	Spinal Cord dorsal column level Th10-11	[24]	CRPS Severity – Phenotype FBSS – CLBP Severity, BMI, PSQI, BDI	SCS chronic stimulation, bipolar, BurstDR vs 40 Hz vs 500 Hz vs 1200 Hz vs sham	ELISA-artificial skin blister (IL-1b, IL-1ra, IL-2, IL-4, IL-5, IL-6, IL-7, IL-8, IL-9, IL-10, IL-12p70, IL-13, IL-15, IL-17, basic FGF, Eotaxin, G-CSF, GM-CSF, IFN-g, IP-10, MCP-1, MIP-1a, MIP-1b, PDGF-BB, TNF-a, VEGF, T cells)	3 months
**2016/pPS [75]**	NP Phantom pain	Spinal Cord dorsal column level Th10-11	[14]	Pain Severity BMI	Chronic stimulation, bipolar, tonic SCS mode with 50 Hz SCS on vs off	Proteomic Mass Spectrometry-CSF (clusterin, gelsolin, mimecan, angiotensinogen, secretogranin-1, amyloid beta A4 protein, gelsolin, apolipoprotein C1, apolipoprotein E, contactin-1, neural cell adhesion molecule L1-like protein, VGF and dickkopf-related protein 3)	12 months
**2014/pPS [77]**	FBSS	Spinal Cord dorsal column level Th10-11	[9]	Pain Severity, SF-36	Chronic stimulation, bipolar, tonic SCS mode with 40–100 Hz, 210–360 µsec, 3–7.4 Volt	ELISA-CSF (GDNF)	
**2013/pPS [76]**	NP Phantom pain	Spinal Cord dorsal column level Th10-11	[14]	Pain Severity, SF-36	Chronic stimulation, bipolar, tonic SCS mode with 40–100 Hz, 210–360 µsec, 3–7.4 Volt	ELISA-CSF (VEGF, BDNF, MCP-1, chemokines)	

CM = chronic migraine; EM = episodic migraine; HFEM = high frequency episodic migraine; FBSS = failed back surgery syndrome; CLBP = chronic lower back pain; CRPS = complex regional pan syndrome; NP = neuropathic pain; VAS = visual analogue scale; HIT = Headache Impact Test; m = month; MIDAS = migraine disability score; BDI = beck depression inventory, PSQI = Pittsburgh Sleep Quality Index; BMI = body mass index; EQl = Quality of life; nVNS = cervical non-invasive vagus nerve stimulation; SCS = tonic spinal cord stimulation; BurstDR-SCS = burst spinal cord stimulation; DRG-SCS = dorsal root ganglion stimulation; CSF = cerebrospinal fluid; IL = interleukines, HMGB-1 = high-mobility group box-1; DAMP´S = damage-associated molecular pattern; free fatty acid receptor 2 (FFAR2); interleukine receptor 1 antagonist (ILRN), interleukine 17-F (IL-17F); phospholipase A2, group IIA (PLA2G2); NADPH oxidase (NOX1); VEGF = vascular endothelial growth faxtor; GDNF = glial cell-derived neurotrophic factor; ELISA = enzyme-linked immune assay; pPS = prospective pilot study; rCS = retrospective case series; RCT = randomized, double-blind sham-controlled study; w = week; y = year.

## References

[1] Breivik H., Collett B., Ventafridda V., Cohen R., Gallacher D. (2006). Survey of chronic pain in Europe: Prevalence, impact on daily life, and treatment. Eur. J. Pain.

[2] Langley P.C., Van Litsenburg C., Cappelleri J.C., Carroll D. (2013). The burden associated with neuropathic pain in Western Europe. J. Med. Econ..

[3] Fishman M.A., Antony A., Esposito M., Deer T., Levy R. (2019). The Evolution of Neuromodulation in the Treatment of Chronic Pain: Forward-Looking Perspectives. Pain Med..

[4] Chaudhry S.R., Lendvai I.S., Muhammad S., Westhofen P., Kruppenbacher J., Scheef L., Boecker H., Scheele D., Hurlemann R., Kinfe T.M. (2019). Inter-ictal assay of peripheral circulating inflammatory mediators in migraine patients under adjunctive cervical non-invasive vagus nerve stimulation (nVNS): A proof-of-concept study. Brain Stimul..

[5] Gravius N., Chaudhry S.R., Muhammad S., Boström A., Gravius S., Randau T., Scheele D., Westhofen P., Kruppenbacher J., Stoffel-Wagner B. (2019). Selective L4 Dorsal Root Ganglion Stimulation Evokes Pain Relief and Changes of Inflammatory Markers: Part I Profiling of Saliva and Serum Molecular Patterns. Neuromodul. Technol. Neural Interface.

[6] Muhammad S., Chaudhry S.R., Yearwood T.L., Krauss J.K., Kinfe T.M. (2018). Changes of Metabolic Disorders Associated Peripheral Cytokine/Adipokine Traffic in Non-Obese Chronic Back Patients Responsive to Burst Spinal Cord Stimulation. Neuromodul. Technol. Neural Interface.

[7] Kinfe T.M., Muhammad S., Link C., Roeske S., Chaudhry S.R., Yearwood T.L. (2017). Burst spinal cord stimulation increases peripheral antineuroinflammatory interleukin 10 levels in failed back surgery syndrome patients with predominant back pain. Neuromodul. Technol. Neural Interface.

[8] Jensen M.P., Hakimian S., Sherlin L.H., Fregni F. (2008). New insights into neuromodulatory approaches for the treatment of pain. J. Pain.

[9] Borowska M., Dworacka M., Wesolowska A., Winiarska H., Krzyzagorska E., Dworacki G. (2016). The impact of pharmacotherapy of type 2 diabetes mellitus on IL-1beta, IL-6 and IL-10 secretion. Pharmacology.

[10] Barry J.C., Shakibakho S., Durrer C., Simtchouk S., Jawanda K.K., Cheung S.T., Mui A.L., Little J.P. (2016). Hyporesponsiveness to the anti-inflammatory action of interleukin-10 in type 2 diabetes. Sci. Rep..

[11] Ma D.H., Xu Q.Y., Liu Y., Zhai Q.Q., Guo M.H. (2016). Association between interleukin-10 gene polymorphisms and susceptibility to diabetic nephropathy in a Chinese population. Genet. Mol. Res..

[12] Dagdeviren S., Jung D.Y., Friedline R.H., Noh H.L., Kim J.H., Patel P.R., Tsitsilianos N., Inashima K., Tran D.A., Hu X. (2017). IL-10 prevents aging-associated inflammation and insulin resistance in skeletal muscle. FASEB J..

[13] Apkarian A.V., Hashmi J.A., Baliki M.N. (2011). Pain and the brain: Specificity and plasticity of the brain in clinical chronic pain. Pain.

[14] Opatrilova R., Caprnda M., Kubatka P., Valentova V., Uramova S., Nosal V., Gaspar L., Zachar L., Mozos I., Petrovic D. (2018). Adipokines in neurovascular diseases. Biomed. Pharm..

[15] Hysing E.B., Smith L., Thulin M., Karlsten R., Bothelius K., Gordh T. (2019). Detection of systemic inflammation in severely impaired chronic pain patients and effects of a multimodal pain rehabilitation program. Scand. J. Pain.

[16] Wåhlén K., Ghafouri B., Ghafouri N., Gerdle B. (2018). Plasma Protein Pattern Correlates with Pain Intensity and Psychological Distress in Women with Chronic Widespread Pain. Front. Psychol..

[17] Gerdle B., Ghafouri B., Ghafouri N., Bäckryd E., Gordh T. (2017). Signs of ongoing inflammation in female patients with chronic widespread pain: A multivariate, explorative, cross-sectional study of blood samples. Medicine (Baltim.).

[18] De La Cruz P., Fama C., Roth S., Haller J., Wilock M., Lange S., Pilitsis J. (2015). Predictors of spinal cord stimulation success. Neuromodulation.

[19] Özaktay A.C., Kallakuri S., Takebayashi T., Cavanaugh J.M., Asik I., Deleo J.A., Weinstein N.J.N. (2006). Effects of interleukin-1 beta, interleukin-6, and tumor necrosis factor on sensitivity of dorsal root ganglion and peripheral receptive fields in rats. Eur. Spine J..

[20] Sorkin L., Xiao W.-H., Wagner R., Myers R. (1997). Tumour necrosis factor-α induces ectopic activity in nociceptive primary afferent fibres. Neuroscience.

[21] Zhang J.-M., Li H., Liu B., Brull S.J. (2002). Acute topical application of tumor necrosis factor α evokes protein kinase A-dependent responses in rat sensory neurons. J. Neurophysiol..

[22] Sun J.H., Yang B., Donnelly D.F., Ma C., Lamotte R.H. (2006). MCP-1 enhances excitability of nociceptive neurons in chronically compressed dorsal root ganglia. J. Neurophysiol..

[23] White F.A., Sun J., Waters S.M., Ma C., Ren D., Ripsch M., Steflik J., Cortright D.N., Lamotte R.H., Miller R.J. (2005). Excitatory monocyte chemoattractant protein-1 signaling is up-regulated in sensory neurons after chronic compression of the dorsal root ganglion. Proc. Natl. Acad. Sci. USA.

[24] Schäfers M., Svensson C.I., Sommer C., Sorkin L.S. (2003). Tumor necrosis factor-α induces mechanical allodynia after spinal nerve ligation by activation of p38 MAPK in primary sensory neurons. J. Neurosci..

[25] Ramer M.S., Murphy P.G., Richardson P.M., Bisby M.A. (1998). Spinal nerve lesion-induced mechanoallodynia and adrenergic sprouting in sensory ganglia are attenuated in interleukin-6 knockout mice. Pain.

[26] Xie W.R., Deng H., Li H., Bowen T., Strong J., Zhang J.M. (2006). Robust increase of cutaneous sensitivity, cytokine production and sympathetic sprouting in rats with localized inflammatory irritation of the spinal ganglia. Neuroscience.

[27] Zingg R.W., Kendall R. (2017). Obesity, vascular disease, and lumbar disk degeneration: Associations of comorbidities in low back pain. PM R.

[28] Samartzis D., Karppinen J., Cheung J.P., Lotz J. (2013). Disk degeneration and low back pain: Are they fat-related conditions?. Glob. Spine J..

[29] Dario A., Ferreira M., Refshauge K., Harmer A., Sanchez-Romera J., Perez-Riquelme F., Cisneros L., Ordonana J., Ferreira P. (2017). Mapping the association between back pain and type 2 diabetes: A cross-sectional and longitudinal study of adult Spanish twins. PLoS ONE.

[30] Samartzis D., Karppinen J., Chan D., Luk K.D., Cheung K.M. (2012). The association of lumbar intervertebral disc degeneration on magnetic resonance imaging with body mass index in overweight and obese adults: A population-based study. Arthritis Rheumatol..

[31] Gupta N., White K.T., Sandford P.R. (2006). Body mass index in spinal cord injury—A retrospective study. Spinal Cord.

[32] Podichetty V.K. (2007). The aging spine: The role of inflammatory mediators in intervertebral disc degeneration. Cell. Mol. Biol..

[33] Vezzani A., Viviani B. (2015). Neuromodulatory properties of inflammatory cytokines and their impact on neuronal excitability. Neuropharmacology.

[34] Srikanthan K., Feyh A., Visweshwar H., Shapiro J.I., Sodhi K. (2016). Systematic review of metabolic syndrome biomarkers: A panel for early detection, management, and risk stratification in the west virginian population. Int. J. Med. Sci..

[35] Bomfim G.F., Rodrigues F.L., Carneiro F.S. (2017). Are the innate and adaptive immune systems setting hypertension on fire?. Pharm. Res..

[36] McMaster W.G., Kirabo A., Madhur M.S., Harrison D.G. (2015). Inflammation, immunity, and hypertensive end-organ damage. Circ. Res..

[37] Crowley S.D. (2014). The cooperative roles of inflammation and oxidative stress in the pathogenesis of hypertension. Antioxid. Redox Signal..

[38] De Ciuceis C., Rossini C., La Boria E., Porteri E., Petroboni B., Gavazzi A., Sarkar A., Rosei E.A., Rizzoni D. (2014). Immune mechanisms in hypertension. High Blood Press. Cardiovasc. Prev..

[39] Golia E., Limongelli G., Natale F., Fimiani F., Maddaloni V., Pariggiano I., Bianchi R., Crisci M., D’Acierno L., Giordano R. (2014). Inflammation and cardiovascular disease: From pathogenesis to therapeutic target. Curr. Atheroscler. Rep..

[40] Fernández-Sánchez A., Madrigal-Santillán E., Bautista M., Esquivel-Soto J., Morales-González Á., Esquivel-Chirino C., Durante-Montiel I., Sánchez-Rivera G., Valadez-Vega C., Morales-González J.A. (2011). Inflammation, oxidative stress, and obesity. Int. J. Mol. Sci..

[41] Kotani K., Sakane N. (2011). Leptin: Adiponectin ratio and metabolic syndrome in the general Japanese population. Korean J. Lab. Med..

[42] Finucane F.M., Luan J., Wareham N.J., Sharp S.J., O’Rahilly S., Balkau B., Flyvbjerg A., Walker M., Hojlund K., Nolan J.J. (2009). Correlation of the leptin: Adiponectin ratio with measures of insulin resistance in non-diabetic individuals. Diabetologia.

[43] Francisco V., Pino J., Gonzalez-Gay M.A., Mera A., Lago F., Gómez R., Mobasheri A., Gualillo O. (2018). Adipokines and inflammation: Is it a question of weight?. Br. J. Pharmacol..

[44] Cong W.N., Golden E., Pantaleo N., White C.M., Maudsley S., Martin B. (2010). Ghrelin receptor signaling: A promising therapeutic target for metabolic syndrome and cognitive dysfunction. CNS Neurol. Disord. Drug Targets.

[45] Varela L., Vazquez M.J., Cordido F., Nogueiras R., Vidal-Puig A., Dieguez C., Lopez M. (2011). Ghrelin and lipid metabolism: Key partners in energy balance. J. Mol. Endocrinol..

[46] Ukkola O. (2009). Ghrelin and metabolic disorders. Curr. Protein Pept. Sci..

[47] Ukkola O. (2005). Ghrelin and the metabolic balance. J. Endocrinol. Investig..

[48] Bacha F., Arslanian S.A. (2005). Ghrelin suppression in overweight children: A manifestation of insulin resistance?. J. Clin. Endocrinol. Metab..

[49] Fagerberg B., Hulten L.M., Hulthe J. (2003). Plasma ghrelin, body fat, insulin resistance, and smoking in clinically healthy men: The atherosclerosis and insulin resistance study. Metabolism.

[50] Farajdokht F., Babri S., Karimi P., Alipour M.R., Bughchechi R., Mohaddes G. (2017). Chronic ghrelin treatment reduced photophobia and anxiety-like behaviors in nitroglycerin-induced migraine: Role of pituitary adenylate cyclase—Activating polypeptide. Eur. J. Neurosci..

[51] Inoue W., Poole S., Bristow A.F., Luheshi G.N. (2006). Leptin induces cyclo-oxygenase 2 via interaction with interleukin 1-beta in the rat brain. Eur. J. Neurosci..

[52] Boström A., Scheele D., Stoffel-Wagner B., Hönig F., Chaudhry S.R., Muhammad S., Hurlemann R., Krauss J.K., Lendvai I.S., Chakravarthy K.V. (2019). Saliva molecular inflammatory profiling in female migraine patients responsive to adjunctive cervical non-invasive vagus nerve stimulation: The MOXY Study. J. Transl. Med..

[53] Ding C., Leow M.K., Magkos F. (2019). Oxytocin in metabolic homeostasis: Implication for obesity and diabetes management. Obes. Rev..

[54] Eliava M.A. (2016). New Population of Parvocellular Oxytocin Neurons Controlling Magnocellular Neuron Activity and Inflammatory Pain Processing. Neuron.

[55] Boll S., Almeida de Minas A.C., Raftogianni A., Herpetz S.C., Grinevich V. (2018). Oxytocin and pain perception: From animal models to human research. Neuroscience.

[56] Poisbeau P., Grinevich V., Charlet A. (2018). Oxytocin Signaling in Pain: Cellular, Circuit, System, and Behavioral Levels. Curr. Top. Behav. Neurosci..

[57] Tzabazis A., Mechanic J., Miller J., Klukinov M., Pascual C., Manering N., Carson D.S., Jacobs A., Qiao Y., Cuellar J. (2016). Oxytocin receptor: Expression in the trigeminal nociceptive system and potential role in the treatment of headache disorders. Cephalalgia.

[58] Tzabazis A., Kori S., Mechanic J., Miller J., Pascual C., Manering N., Carson D., Klukinov M., Spierings E., Jacobs D. (2017). Oxytocin and Migraine Headache. Headache.

[59] Hoshiyamata E., Tatsumoto M., Iwanami H., Saisu A., Watanabe H., Inaba N., Hirata K. (2012). Postpartum migraines: A long-term prospective study. Intern. Med..

[60] Phillips W.J., Ostrovsky O., Galli R.L., Dickey S. (2006). Relief of acute migraine headache with intravenous oxytocin: Report of two cases. J. Pain Palliat. Care Pharmacother..

[61] Noseda R., Lee A.J., Rony-Reuven N., Bernstein C.A., Kainz V.M., Bertisch S.M., Buettner C., Borsook D., Burstein R. (2017). Neural mechanism for hypothalamic-mediated autonomic responses to light during migraine. Proc. Natl. Acad. Sci. USA.

[62] Strother L.C., Srikiatkhachorn A., Supronsinchai W. (2018). Targeted orexin and hypothalamic neuropeptides for migraine. Neurotherapeutics.

[63] You D.S., Honey R., Albu S., Meagher M.W. (2018). Generalized pain sensitization and endogenous oxytocin in individuals with symptoms of migraine: A cross-sectional study. Headache.

[64] Martínez-Lorenzana G., Espinosa-López L., Carranza M., Aramburo C., Paz-Tres C., Rojas-Piloni G., Condés-Lara M. (2008). PVN electrical stimulation prolongs withdrawal latencies and releases oxytocin in cerebrospinal fluid, plasma, and spinal cord tissue in intact and neuropathic rats. Pain.

[65] Condés-Lara M., Maie I.A., Dickenson A.H. (2005). Oxytocin actions on afferent evoked spinal cord neuronal activities in neuropathic but not in normal rats. Brain Res..

[66] Breton J.D., Veinante P., Uhl-Bronner S., Vergnano A.M., Freund-Mercier M.J., Schlichter R., Poisbeau P. (2008). Oxytocin-induced antinociception in the spinal cord ismediated by a subpopulation of glutamatergic neurons in lamina I-II which amplify GABAergic inhibition. Mol. Pain.

[67] Condés-Lara M., Rojas-Piloni G., Martínez-Lorenzana G., López-Hidalgo M., Rodríguez-Jiménez J. (2009). Hypothalamospinal oxytocinergic antinociception ismediated by GABAergic and opiate neurons that reduce Adelta and C fiber primary afferent excitation of spinal cord cells. Brain Res..

[68] Moreno-López Y., Martinez-Lorenzana G., Condés-Lara M., Rojas-Piloni G. (2013). Identification of oxytocin receptor in the dorsal dorn and nociceptive dorsal root ganglion neurons. Neuropeptides.

[69] Tracey K.J. (2002). The inflammatory reflex. Nature.

[70] Pavlov V.A., Tracey K.J. (2012). The vagus nerve and the inflammatory reflex—Linking immunity and metabolism. Nat. Rev. Endocrinol..

[71] Stock S., Uvnas-Moberg K. (1988). Increased plasma levels of oxytocin in response to electrical stimulation of the sciatic and vagal nerves and in response to touch and pinch in anaesthetized rats. Acta Physiol. Scand..

[72] Berthoud H.R., Neuhuber W.L. (2019). Vagal mechanisms as neuromodulatory targets for the treatment of metabolic disease. Ann. N. Y. Acad. Sci..

[73] Corcoran C., Connor T.J., O´Keane V., Garland M.R. (2005). The effects of vagus nerve stimulation on pro- and anti-inflammatory cytokines in Humans: A preliminary report. Neuroimmunomodulation.

[74] Chakravarthy K.V., Xing F., Bruno K., Kent A.R., Raza A., Hurlemann R., Kinfe T.M. (2019). A Review of Spinal and Peripheral Neuromodulation and Neuroinflammation: Lessons Learned Thus Far and Future Prospects of Biotype Development. Neuromodul. Technol. Neural Interface.

[75] Lind A.-L., Khoonsari E., Sjodin M., Katila L., Wetterhall M., Gordh T., Kultima K. (2016). Spinal cord stimulation alters protein levels in the cerebrospinal fluid of neuropathic pain patients: A proteomic mass spectrometric analysis. Neuromodul. Technol. Neural Interface.

[76] McCarthy K.F., Connor T., McCrory C. (2013). Cerebrospinal fluid levels of vascular growth factor correlate with reported pain and are reduced by spinal cord stimulation in patients with failed back surgery syndrome. Neuromodul. Technol. Neural Interface.

[77] McCarthy K.F., McCrory C. (2014). Cerebrospinal fluid levels of glial cell-derived neurotrophic factor correlate with spinal cord stimulation frequency in patients with neuropathic pain: A preliminary report. Spinal Cord.

[78] Kriek N., Schreurs M.W.J., Groeneweg J.G., Dik W.A., Tjiang G.C.H., Gültuna I., Stronks D.L., Huygen F.J.P.M. (2018). Spinal Cord Stimulation in Patients with Complex Regional Pain Syndrome: A Possible Target for Immunomodulation?. Neuromodul. Technol. Neural Interface.

[79] Kinfe T.M., Asif M., Chakravarthy K.V., Deer T.R., Kramer J.M., Yearwood T.L., Hurlemann R., Hussain M.S., Motameny S., Wagle P. (2019). Unilateral L4-dorsal root ganglion stimulation evokes pain relief in chronic neuropathic postsurgical knee pain and changes of inflammatory markers: Part II whole transcriptome profiling. J. Transl. Med..

[80] Lerman I., Hauger R., Sorkin L., Proudfoot J., Davis B., Huang A., Lam K., Simon B., Baker D.G. (2016). Noninvasive Transcutaneous Vagus Nerve Stimulation Decreases Whole Blood Culture-Derived Cytokines and Chemokines: A Randomized, Blinded, Healthy Control Pilot Trial. Neuromodul. Technol. Neural Interface.

[81] Russo M.A., Fiore N.T., van Vreden C., Bailey D., Danielle M.S., Helen M.M., de St Groth B.F., Austin P.J. (2019). Expansion and activation of distinct central memory T lymphocyte subsets in complex regional pain syndrome. Austin J. Neuroinflamm..

[82] Dahlem M.A., Rode S., May A., Fujiwara N., Hirata Y., Kazuyuki A., Kurths J. (2013). Towards dynamical network biomarkers in neuromodulation of episodic migraine. Transl. Neurosci..

